# Association between Accreditation and In-Hospital Mortality in Patients with Major Cardiovascular Diseases in South Korean Hospitals: Pre-Post Accreditation Comparison

**DOI:** 10.3390/medicina56090436

**Published:** 2020-08-28

**Authors:** You Jin Chun, Bo Yeon Lee, Yo Han Lee

**Affiliations:** 1 Korea Institute for Healthcare Accreditation, Seoul 07238, Korea; ugenuin@naver.com; 2 Health Insurance Review and Assessment Service, Wonju 26465, Korea; tarasyn99@korea.ac.kr; 3 Graduate School of Public Health, Ajou University, Suwon 16499, Korea

**Keywords:** accreditation, acute myocardial infarction, ischemic stroke, hemorrhagic stroke, 30-day mortality

## Abstract

The direct impact of hospital accreditation on patients’ clinical outcomes is unclear. The purpose of this study was to evaluate whether mortality within 30 days of hospitalization for acute myocardial infarction (AMI), ischemic stroke (IS), and hemorrhagic stroke (HS) differed before and after hospital accreditation. This study targeted patients who had been hospitalized for the three diseases at the general hospitals newly accredited by the government in 2014. Thirty-day mortality rates of three years before and after accreditation were compared. Mortality within 30 days of hospitalization for the three diseases was lower after accreditation than before (7.34% vs. 6.15% for AMI; 4.64% vs. 3.80% for IS; and 18.52% vs. 15.81% for HS). In addition, hospitals that meet the criteria of the patient care process domain have a statistically lower mortality rate than hospitals that do not. In the newly accredited Korean general hospital, it was confirmed that in-hospital mortality rates of major cardiovascular diseases were lower than before the accreditation.

## 1. Introduction

Hospital accreditation is widely recognized as a tool to improve the health care system and evaluate the quality of health [1,2], and is being implemented in many countries around the world as an effective strategy to ensure and improve the quality of healthcare services [3,4,5,6]. Hospital accreditation was initiated in 1917 by the American College of Surgeons, and since then the number of hospital accreditation programs has increased rapidly [7]. Hospital licensure requires governments to comply with minimum standards to ensure patient safety, while hospital accreditation is typically performed by non-governmental organizations, voluntary participation by hospitals, and the highest standards [8].

According to existing literature on hospital accreditation, it has positive effects such as establishment of organizational structure and processes, improvement of quality and safety culture, improvement of patient care, and development of professionalism [9,10,11]. A systematic review of the effectiveness of accreditation found that there were positive aspects of accreditation, but this study did not provide a solid basis to support that conclusion [12]. Evidence has also been reported that accreditation is rather negative. In terms of reducing clinical learning opportunities and increasing non-medical workloads, there was a negative impact of accreditation on the learning environment of medical students and trainees [13].

However, most of the existing research on the impact of accreditation was on the structural factors or care processes of hospitals. Few studies have investigated whether accreditation has a positive effect on patient outcomes such as death or readmission, and the results have also been mixed [14,15,16,17]. The purpose of this study is to evaluate whether the 30-day mortality rate in hospitalization of patients with acute myocardial infarction (AMI), ischemic stroke (IS), and hemorrhagic stroke (HS) admitted to general hospitals in Korea is different before and after hospital accreditation.

## 2. Materials and Methods

### 2.1. Data Sources and Study Population

Since Korea has a health insurance system operated by a single insurer, researchers can conduct meaningful outcome studies through data on the use of medical care by almost all citizens [18]. South Korea has also been conducting hospital accreditation at each level through an independent non-government agency (Korea Institute for Healthcare Accreditation, KIHA, Seoul, Korea), which comprehensively evaluates the rights and safety of patients, activities to improve the quality of medical services, the process and performance of medical services, the hospitals’ manpower management and operation, and patient satisfaction [19]. The evaluation criteria of the KIHA hospital accreditation program consist of a total of four domains: basic value, patient treatment, administrative, and performance management. Of these, the basic value, administrative, and performance management domains consisted of a total of 44 indicators addressing the structure and administrative aspects of the hospital. The patient care domain, a key component of accreditation, consisted of 47 indicators that measure the patient care process [20]. The hospital-level accreditation system currently operating in Korea is only for training hospitals. Non-training hospitals are excluded from accreditation.

This study targeted general hospitals in Korea, which were newly accredited by KIHA in 2014. The claims data for patients admitted to these hospitals with AMI (I21), IS (I63), and HS (I60-I62) from 2010 to 2017 were obtained from the National Health Insurance Corporation. A total of 183 general hospitals, all of which were teaching hospitals, and 248,630 patients were included. Data on the deaths of these patients within 30 days of admission were obtained from the National Statistical Office. The protocol of this study was approved by the Institutional Review Board of Korea University (IRB No. KUIRB-2018-0095-01).

### 2.2. Variables and Definitions

The claims data have both patient variables and hospital variables. Patient variables include sex, age, type of health coverage (health insurance vs. medical aid), degree of comorbidity, and hospitalization path (via emergency room vs. planned hospitalization). For risk adjustment, the level of comorbidity that best reflects the patient’s clinical condition was determined using the Charlson Comorbidity Index (CCI index) [21] and was classified into three categories according to the CCI index (no comorbidity: 0 points, low comorbidity: 1 to 2 points, high comorbidity: 3 points or more). CCI is a method of categorizing patients’ comorbidities based on the International Classification of Disease (ICD) diagnosis. In this study, 13 comorbidities commonly calculated for Koreans were selected as follows [22]: diabetes mellitus, congestive health failure, peripheral vascular disease, dementia, chronic pulmonary disease, rheumatic disease, gastric/peptic ulcer, mild liver disease, hemiplegia/paraplegia, renal disease, any malignancy, metastatic solid tumor, and acquired immune deficiency syndrome.

Hospital variables included the number of medical personnel, hospital ownership, and the area where the hospital was located. The number of doctors and nurses is expressed as a number per 100 beds. Hospital ownership was divided into public, corporate, and individual. The location of the hospital was divided into the metropolitan area and the non-metropolitan area.

### 2.3. Analyses

First, the study subjects were divided into two groups: patients for 3 years before accreditation and patients for 3 years after accreditation. Second, 30-day mortality following admission for the three diseases in these two groups was compared. Third, the multiple logistic regression analysis was performed to determine whether the satisfaction of each accreditation domain was related to the mortality rate of the three diseases. The achievement of each domain was based on the criteria set by KIHA, that is, 80% or more of the indicators of each domain were satisfied. The basic value domain was excluded from this analysis because almost all hospitals met the basic value domain. Due to the nature of the analysis, this last analysis was conducted with data from three years after accreditation. For data processing and all statistical analysis, SAS 9.4 was used (version 9.3, SAS Institute Inc., Cary, NC, USA).

## 3. Results

Table 1 shows the characteristics of patients with AMI, IS and HS, and hospitals before and after accreditation. The distribution of characteristics between the two groups are similar. Table 2 shows the mortality rate of 30 days of hospitalization for each 3 years before and after accreditation. In all three diseases, the 30-day mortality rates at each three-year period after accreditation were lower than all the 30-day mortality rates at each three-year period before accreditation.

**Figure 1:**
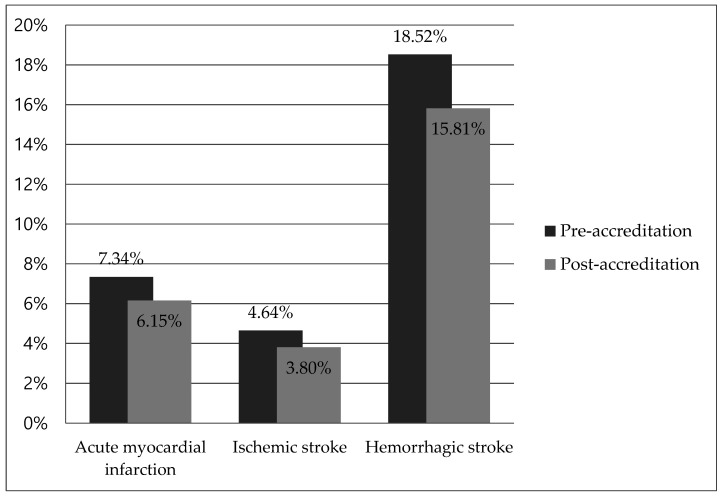
Thirty-day mortality rates between pre- and post-accreditation. All differences were statistically significant.

Mortality rates within 30 days of admission between the two groups before and after accreditation are shown in Figure 1. The mortality rate of AMI patients during the three years before accreditation was 7.34%, whereas the post-accreditation mortality was 6.15%. Similar results were seen in patients with IS and HS. The pre- and post-accreditation mortality rates for IS and HS were 4.64% and 3.80%, 18.52% and 15.81%, respectively.

Table 3 shows the relationship between the achievement of each accreditation domain and the mortality rate of the three diseases through multiple logistic regression analysis. Hospitals that achieved patient care domain tended to have lower mortality rates than hospitals that did not. In particular, the odds ratio for AMI and HS was statistically significant (0.162 and 0.354, respectively). Achieving the performance management domain also showed some relevance to the low mortality rates. On the other hand, the achievement of the administrative domain was not significantly related to the mortality rates.

## 4. Discussion

### 4.1. Main Findings

Accumulating evidence partially suggests the beneficial effects of accreditation on care processes and patient outcomes, but it remains uncertain if accreditation has a direct impact on patient mortality. This study investigated whether the ultimate goal of hospital accreditation programs, that is, improving patient outcomes, is actually being achieved through major diseases, clear indicator and intuitive study design. The key finding of this study was that the mortality rate within 30 days of hospitalization due to AMI and cerebral stroke was significantly reduced after accreditation compared to before. In addition, the fact that hospitals meeting the criteria of the patient care process domain, the key domain of accreditation, had lower mortality rates than hospitals that did not, suggests a mechanism to explain the difference in mortalities between before and after accreditation. Since this patient care process domain includes various aspects related to patient care such as treatment delivery, patient evaluation, diagnostic testing, and surgery and procedures, it is highly likely that quality improvement in this domain has reduced patient mortality. This result was from the same hospitals, and there was no significant change in the medical workforce level or patient characteristics before and after accreditation.

Since this study covers all hospitals and universities in Korea, which were newly accredited in 2014, our findings can be generalized in the Korean context. Previous studies that have seen the effect of hospital accreditation on mortality in Korea have never been known. The results of this study, although based on a simple analysis, are likely to help in the operation of hospital accreditation programs that have been criticized for lack of evidence for its effectiveness in Korea.

### 4.2. Strengths of This Study

Many studies exploring the relationship between accreditation and clinical outcomes have usually used a design that compares the outcomes of accredited and non-accredited hospitals, and there is considerable room for various unknown confounders. For example, there is the potential of selection bias in terms of which hospitals have decided to be accredited. Given that accreditation is an option, one would assume that hospitals with more resources will go through this process [23]. The main strength of our study is that this kind of selection bias was fundamentally prevented through pre- and post-accreditation comparisons of the same hospitals.

AMI and cerebral stroke are well suited for a study of accreditation as they are common diagnoses and major causes of morbidity and mortality for which quality measures have been established [17]. Mortality is one of the most easily understood outcomes of healthcare. Unlike many other constructs, such as quality of life or functional status, death is unambiguous, clearly defined and universally resonant for patients, clinicians and managers. The rationale for measuring in-hospital 30-day mortality is that deaths after a longer time period may have less to do with the care the hospital provided and more to do with other complicating illnesses, patients’ own behavior, or other care services patients received after they leave the hospital. Measuring and reporting of 30-day mortality after hospitalization for the major cardiovascular diseases is a widely used measure of hospital performance across countries. In Norway, in-hospital 30-day mortality due to stroke and acute myocardial infarction has been reported annually as an indicator of care quality for all hospitals [24,25,26].

### 4.3. Comparison with Previous Studies

Unfortunately, it is difficult to make a meaningful direct comparison with previous studies since to the best of our knowledge, there are no studies like our study which is (i) targeting AMI and stroke, (ii) comparing before and after accreditation, and (iii) using mortality as an outcome variable at the same time. This difficulty is even greater considering that the effect of accreditation can vary from disease to disease [27]. Nevertheless, a careful comparison of previous studies with our findings was performed as follows. A study by Falstie-Jensen A.M. et al. found that high-compliance hospitals that were serially accredited had significantly lower risk of death within 30 days of hospitalization compared to low-compliance hospitals that were conditionally accredited. As in our study, these results indicate the relationship between accreditation and low patient mortality [28]. However, this study differs from our study in that it included 80 diseases that accounted for 80% of the causes of death within 30 days of hospitalization.

Bogh SB et al.’s studies that conducted comparisons before and after accreditation, showed that process performance measures for acute stroke, heart failure, gastric ulcer, diabetes, breast cancer, and lung cancer were improved after accreditation than before [29,30]. This is in line with the results of previous studies that hospital accreditation improves the care process, providing a reasonable mechanism for our findings that accreditation reduces mortality. On the other hand, Devkaran S. et al.’s studies which have used interrupted time series analysis to see if there were significant differences in the clinical quality measures before and after accreditation for all diseases reported that the structure and process quality indicators improved after accreditation compared to before, but the patient mortality reduction was not significant [31,32]. Given that the effect of accreditation on the mortality reduction can be disease-specific [27], it is natural that these studies targeting all diseases did not clearly demonstrate the effect of accreditation on patient mortality reduction. In addition to these, the results of other studies using mortality as an outcome indicator reported results in which accreditation had little or no effect on mortality reduction [16,33,34].

Combining these previous studies, it can be seen that the evidence that accreditation reduces hospital mortality is not so clear. In contrast, in our study, the mortality rates after accreditation for AMI, IS, and HS decreased by about 19%, 22%, and 17%, respectively, compared to before accreditation. Careless conclusions about the causality of our study should be avoided because there is insufficient evidence to explain or support the relatively large reduction in mortality before and after accreditation.

### 4.4. Limitations of This Study

Our study has several limitations. First, a more complete level of risk adjustment was not performed for mortality within 30 days of admission. In many countries where medical quality assessment is performed, risk-standardized mortality rates which require complex statistical calculations are used for specific diseases [35,36]. However, we performed some degree of risk adjustment through an academically recognized method for reflecting co-morbidity (CCI index), which is the core of risk adjustment. In addition, multiple logistic regression analysis provides more meaningful results because of controlling various factors that could affect mortality rate. Above all, the fact that the characteristics of the study subject and the hospital did not show much difference before and after accreditation provides a certain degree of comparability between the two groups.

Second, the design of this study, a pre- and post-accreditation comparison, has significant limitations in demonstrating the mechanisms of change in patient mortality. We have previously mentioned the possibility that mortality changes may have resulted from improved patient care processes. However, the results of our study cannot completely rule out the possibility of any factors that authors did not think of, not accreditation. Therefore, future studies require a longitudinal design the same hospital measuring more than three times in a time series. In addition, a comparison between accredited hospitals and non-accredited hospitals needs to be made.

Third, it should be considered that factors that are not covered by accreditation, such as whether the patient received rehabilitation services or how the treatment type has changed, may also affect the patient’s clinical outcome. Several studies in Korean have reported that rehabilitation services for major cardiovascular disease improve patient clinical outcomes [37,38,39]. Therefore, it is necessary to be cautious in interpreting the results of this study too strongly as causality between the relationship between certification and mortality.

## 5. Conclusions

This study investigated whether mortality within 30 days of hospitalization for AMI, IS, and HS differed before and after hospital accreditation. In the newly accredited Korean general hospitals, 30-day mortality rates of these major cardiovascular diseases were significantly lower than before the accreditation. These results suggest, to some extent, the effectiveness of hospital accreditation, along with the fact that meeting the patient care domain is associated with a low mortality rate for the diseases. However, these results should be handled with caution because there is insufficient evidence to establish a causal relationship.

## Figures and Tables

**Table 1:** General characteristics of study subjects by pre- and post-accreditation.

		Acute Myocardial Infarction				Ischemic Stroke				Hemorrhagic Stroke			
		Pre Accreditation *n* = 20,512		Post Accreditation *n* = 26,766		Pre Accreditation *n* = 64,553		Post Accreditation *n* = 79,034		Pre Accreditation *n* = 26,602		Post Accreditation *n* = 31,202	
**Patient**													
Sex	Male	15,677	(76.43)	20,919	(78.16)	38,903	(60.27)	48,233	(61.03)	15,124	(56.85)	17,435	(55.88)
	Female	4835	(23.57)	5847	(21.84)	25,650	(39.73)	30,801	(38.97)	11,478	(43.15)	13,767	(44.12)
Age	<50	2235	(10.90)	3701	(13.83)	4074	(6.31)	6251	(7.91)	5189	(19.51)	6881	(22.05)
	50–64	8048	(39.24)	11,061	(41.32)	18,940	(29.34)	24,914	(31.52)	10,365	(38.96)	12,557	(40.24)
	65+	10,229	(49.87)	12,004	(44.85)	41,539	(64.35)	47,869	(60.57)	11,048	(41.53)	11,764	(37.70)
Insurance Type	Medical aid	1522	(7.42)	1667	(6.23)	7590	(11.76)	7399	(9.36)	3272	(12.30)	2932	(9.40)
	Insurance	18,990	(92.58)	25,099	(93.77)	56,963	(88.24)	71,635	(90.64)	23,330	(87.70)	28,270	(90.60)
Charlson comorbidity index	0	16,642	(81.13)	21,723	(81.16)	47,335	(73.33)	59,514	(75.30)	20,859	(78.41)	24,868	(79.70)
	1	3658	(17.83)	4781	(17.86)	14,656	(22.70)	16,790	(21.24)	4584	(17.23)	5186	(16.62)
	2	212	(1.03)	262	(0.98)	2562	(3.97)	2730	(3.45)	1159	(4.36)	1148	(3.68)
Admission Type	via emergency room	13,594	(66.27)	18,014	(67.30)	30,263	(46.88)	38,048	(48.14)	16,646	(62.57)	19,814	(63.50)
	via outpatient care	6918	(33.73)	8752	(32.70)	34,290	(53.12)	40,986	(51.86)	9956	(37.43)	11,388	(36.50)
**Hospital**													
Workforce per 100 beds	No. of physician	31.91		31.98		29.07		28.89		32.07		31.54	
	No. of nurses	88.38		88.63		80.93		80.97		85.34		85.04	
Ownership	Public	2121	(10.34)	2468	(9.22)	8770	(13.59)	9988	(12.64)	3355	(12.61)	3712	(11.90)
	Corporate	17,154	(83.63)	22,636	(84.57)	50,853	(78.78)	61,716	(78.09)	21,364	(80.31)	24,705	(79.18)
	Individual	1237	(6.03)	1662	(6.21)	4930	(7.64)	7330	(9.27)	1883	(7.08)	2785	(8.93)
Region	Metropolitan	9298	(45.33)	12,847	(48.00)	29,829	(46.21)	36,559	(46.26)	13,361	(50.23)	15,349	(49.19)
	Nonmetropolitan	11,214	(54.67)	13,919	(52.00)	34,724	(53.79)	42,475	(53.74)	13,241	(49.77)	15,853	(50.81)

Note: brackets: percentages; public ownership: hospitals owned by central or local governments; corporate ownership: hospitals owned by non-profit organizations; individual ownership: hospital owned by an individual.

**Table 2:** Thirty-day mortality for three years before and after accreditation.

	30-Day Mortality before Accreditation			30-Day Mortality after Accreditation		
	3 Years before	2 Years before	1 Year before	1 Year after	2 Years after	3 Years after
Acute myocardial infarction	510/6730 (7.58%)	485/6566 (7.39%)	530/7216 (7.34%)	523/7997 (6.54%)	488/8646 (5.64%)	635/10,123 (6.27%)
Ischemic stroke	880/19,209 (4.58%)	1064/22,231 (4.79%)	1050/23,113 (4.54%)	1011/24,317 (4.16%)	947/25,939 (3.65%)	1049/28,778 (3.65%)
Hemorrhagic stroke	1643/8010 (20.51%)	1661/9261 (17.94%)	1624/9331 (17.40%)	1654/9545 (17.33%)	1603/10,177 (15.75%)	1677/11,480 (14.61%)

**Table 3:** Multiple logistic regression analyses for 30-day mortality by three evaluation domains of hospital accreditation program.

	Acute Myocardial Infarction		Ischemic Stroke		Hemorrhagic Stroke	
Domain	Adjusted Odds Ratio *	95% Confidence Interval	Adjusted Odds Ratio *	95% Confidence Interval	Adjusted Odds Ratio *	95% Confidence Interval
Achieving the patient care domain (vs. not achieving)	0.162	0.035–0.760	0.539	0.186–1.559	0.354	0.128–0.983
Achieving the administrative domain (vs. not achieving)	0.802	0.369–1.744	1.444	0.882–2.364	1.331	0.846–2.095
Achieving the performance management domain (vs. not achieving)	0.540	0.299–0.975	0.975	0.576–1.652	0.529	0.360–0.779

Note: the achievement of each domain was based on the criteria set by Korea Institute for Healthcare Accreditation, that is, 80% or more of the indicators of each domain were satisfied; * adjusted for sex, age, insurance type, comorbidity, admission type, health workforce, and hospital ownership and region.
